# Magnetic Nanoparticles Attached to the NK Cell Surface for Tumor Targeting in Adoptive Transfer Therapies Does Not Affect Cellular Effector Functions

**DOI:** 10.3389/fimmu.2019.02073

**Published:** 2019-08-30

**Authors:** Laura Sanz-Ortega, José M. Rojas, Yadileiny Portilla, Sonia Pérez-Yagüe, Domingo F. Barber

**Affiliations:** Department of Immunology and Oncology, and NanoBiomedicine Initiative, Centro Nacional de Biotecnología (CNB)-CSIC, Madrid, Spain

**Keywords:** cell-based therapy, NK cell, magnetic nanoparticle, magnetic retention, cancer immunotherapy

## Abstract

Adoptive cell transfer therapy is currently one of the most promising approaches for cancer treatment. This therapy has some limitations, however, such as the dispersion of *in vivo*-administered cells, causing only a small proportion to reach the tumor. Nanotechnological approaches could offer a solution for this drawback, as they can increase cell retention and accumulation in a region of interest. In particular, strategies employing magnetic nanoparticles (MNPs) to improve targeting of adoptively transferred T or NK cells have been explored in mice. *In vivo* magnetic retention is reported using the human NK cell line NK-92MI transfected with MNPs. Primary NK cells are nonetheless highly resistant to transfection, and thus we explore in here the possibility of attaching the MNPs to the NK cell surface to overcome this issue, and examine whether this association would affect NK effector functions. We assessed the attachment of MNPs coated with different polymers to the NK cell surface, and found that APS-MNP attached more efficiently to the NK-92MI cell surface. In association with MNPs, these cells preserved their main functions, exhibiting a continued capacity to degranulate, conjugate with and lyse target cells, produce IFN-γ, and respond to chemotactic signals. MNP-loaded NK-92MI cells were also retained in an *in vitro* capillary flow system by applying an EMF. A similar analysis was carried out in primary NK cells, isolated from mice, and expanded *in vitro*. These primary murine NK cells also maintained their functionality intact after MNP treatment and were successfully retained *in vitro*. This work therefore provides further support for using MNPs in combination with EMFs to favor specific retention of functional NK cells in a region of interest, which may prove beneficial to adoptive cell-therapy protocols.

## Introduction

Cancer is one of the leading causes of death in our society, and developing successful therapies to halt cancer progression is a central goal around the world. Currently, immunotherapy, and in particular adoptive transfer therapy, is one of the most promising approaches to treat cancer. NK cells play an essential role in host immunity to cancer and as such are ideal candidates for adoptive transfer. First identified in the 1970s by Kiessling, NK cells are a subpopulation of lymphocytes that are larger in size than T and B lymphocytes and are capable of eliminating tumor cells without prior sensitization (1–3).

Resting NK cells constitute 10–15% of the circulating lymphocytes in peripheral blood, and activation by cytokines and chemokines induces the extravasation and recruitment of these cells to tumor or inflamed tissues (4). This activation of NK effector functions is modulated by a balance between signals transmitted by the inhibitory and activating receptors present on the cell surface. NK cells remain inactive when they encounter normal or healthy cells that express MHC class I molecules, which interact with the inhibitory receptors, and barely express ligands for NK-activating receptors. However, their cytotoxic potential is released when a malignant cell shows an altered or reduced MHC class I profile together with high expression of activating ligands (5). NK cells can also modulate the activity of different immune-cell types within the tumor microenvironment by direct contact or through the release of cytokines or chemokines (6), such as IFN-γ or TNFα, that can amplify and recruit an inflammatory response through various mechanisms (7, 8). They are a major source of IFN-γ *in vivo*, considered crucial to inhibit tumor angiogenesis and in the modeling of adaptive immunity (9, 10).

There is ample evidence supporting the importance of NK cells in antitumor immunity. NK cell depletion in murine tumor models of allogeneic transplantation has established the crucial role of NK cells in the control of tumor growth and metastasis (11, 12). NK cell infusions into mice lacking this cell type generated resistance against the formation of metastasis (12). Allogenic or autologous human NK cells can recognize and eliminate patient biopsy-derived tumor cells *in vitro*. High levels of intratumoral NK cells correlate with greater survival in several malignancies such as colorectal, gastric, or esophageal cancer (13–16). Tumor cell recognition is likely based on a combination of NK cell activation by activating receptor (NKG2D, DNAM-1, NKp30, NKp44), a low expression of MHC class I on tumor cells, and mismatched KIR ligands for the graft vs. hematological malignancy effects (14–17). An 11-year prospective study has correlated the high and moderate cytotoxic activity of peripheral blood NK cells with a lower risk of developing cancer, providing further evidence of the role of NK cells in tumor immunovigilance (17). These studies highlight the beneficial role of NK cells in cancer prognosis and their potential use to treat cancer.

Since NK cells do not depend on the existence of a tumor antigen or previous activation, and are easy to obtain through peripheral blood, they represent an attractive reagent for immunotherapy. NK cells speed of action, power, broad specificity, the absence of side effects, and the fact that they can complement other therapeutic approaches such as chemo/radiotherapy make them particularly suited for adoptive therapy. Ruggeri et al. demonstrated the potent clinical effect of alloreactive NK cells in patients with AML (18), while Miller et al. demonstrated that transfer of haploidentical NK cells expanded in patients with advanced cancer together with subcutaneous IL-2 is a safe treatment (19).

Adoptive transfer of NK cells requires a large number of cells, which are usually obtained from peripheral blood and expanded *ex vivo*. NK cells can also be generated from haematopoietic stem cells from the umbilical cord (20) or from embryonic or pluripotent stem cells (21). Furthermore, the use of purified NK cell lines such as the NK-92 line, which is derived from clones of malignant NK cells, is being explored widely. The NK-92 cell line is an IL-2-dependent NK human cell line that was established in 1992 using the peripheral blood of a non-Hodgkin's lymphoma patient (22, 23). This human NK cell line is characterized by the expression of CD56 and the absence of CD16, and therefore, of ADCC. NK-92 cells lack almost all KIR inhibitory receptors (24) and express the activating receptors NKG2D, NKp30 and NKp46, but not NKG2C or NKp44 (24, 25). This NK cell line has superior cytotoxicity against multiple targets compared to primary or traditional NK cells (23, 26–28), probably due to the apparent lack of inhibitory receptors and the presence of high levels of perforin and granzyme B. Its cytotoxicity has been demonstrated both *in vitro* (23) and *in vivo* in human melanoma and leukemia xenotransplants (26, 27). In addition, this cell line is attracting much attention due to the ease with which it can be cultured and genetically modified when compared to primary NK cells. For instance, NK-92 cell modification with CARs are being explored as routes to overcome escape mechanisms and redirect more specifically their NK cell activity (29, 30). Several ongoing clinical trials have already proved NK-92 safety in these settings (31).

In spite of its promise, NK cell adoptive transfer has only achieved modest results at the clinical level (32). Transferred autologous NK cells can occasionally express low levels of activation markers or activating receptors such as NKG2D. Additionally, *in vivo*-administered NK cells tend to disperse throughout the organism such that only a small proportion reach the tumor. The combination of these two factors makes for inefficient tumor elimination (33). Therefore, much effort is being made to understand the molecular mechanisms that govern the maturation of NK cells and their effector activity against tumor cells, which will help to design optimal protocols for the therapeutic application of NK cells (34). Similarly, the design of strategies that facilitate accumulation of NK cells in the tumor area will enable the development of more efficient antitumor therapies (35). Nanotechnology has proven very useful in selectively directing drugs or molecules to a target site, and this knowledge could be used to develop more effective treatment protocols. One promising strategy consists of magnetically targeting these administered NK cells through the combination of MNPs and EMFs so they may be concentrated in the region of interest. This approach has been used to accumulate stem cells, mesenchymal cells, macrophages, and dendritic cells in tissue-regeneration therapies and for the treatment of autoimmune disorders (29, 36–40), though it has seen very limited application in redirecting lymphoid cells such as T or NK cells to treat cancer (29, 30). Indeed, there is only one report in which MNPs were magnetically transfected into the human NK-92MI cell line, allowing tumor-targeting of MNP-loaded NK cells using an external magnet (30). Primary NK cells are notoriously difficult to transfect and typically require cytokine stimulation or pattern recognition receptor (PRR) signaling inhibition to be effective (41, 42), suggesting that MNP loading through magnetic transfection could be problematic in primary cells. Previous results in our lab have shown that APS-MNPs can be attached to the cytoplasmic membrane of primary murine T cells, and that these nanoparticles enable specific targeting and retention of the modified T cells to lymph nodes using magnets (43).

To determine whether specific magnetic retention of completely functional NK cells could be achieved, we assess magnetic nanoparticles attachment to the NK cell surface and the targeting and retention of the modified NK cells through the application of an EMF. We also examine whether this modification alters functional aspects of NK cell biology. To do this, we first used the prototypic NK cell line NK-92MI, which had been magnetically transfected in another report (30), to evaluate the feasibility of attaching nanoparticles on the surface of cells instead of transfecting them. NK-92MI cells are derived from NK-92 cells, which originated from a patient with granular lymphoma and displays great cytotoxicity for a variety of tumor lines (44–46), including the human K562 cell line (47). NK-92 cells are the only NK cell line approved by the FDA for use in clinical trials and was shown to be safe to use as cell transfer in humans (26, 48, 49). NK-92MI cells were obtained by transfection of the IL-2-encoding gene, and as a result their growth is independent of IL-2 (50, 51). This variant is identical to parental cells in nearly all aspects and their use could allow prolonged treatment with active NK cells without the necessity for exogenous IL-2 (50, 51).

We also carried out a similar analysis in primary murine NK cells as a model. The results showed that the human NK-92MI cell line and the primary murine NK cells preserved their functionality in the presence of MNP attached to the cell surface. Both NK cell models loaded with MNPs were also retained in an *in vitro* capillary flow system by using a magnet.

This work details an interesting and simple approach which could be used to improve NK cell migration to a region, thereby increasing the number of cytolytic NK cells with intact functionality that reach the tumor, leading to more efficient treatment.

## Materials and Methods

### MNP Synthesis and Physico-Chemical Characterization

The synthesis and characterization of the different MNPs used in this study have been described previously (43). Briefly, iron-oxide cores were synthesized by following the Massart co-precipitation protocol (52), and these iron cores were then coated with dimercaptosuccinic acid (DMSA), (3-aminopropyl) triethoxysilane (APS), or dextran 6 kDa (DEXT) in accordance with the previously described procedures (53). Next, we performed a physico-chemical characterization of the different coated MNPs. The hydrodynamic diameter and Z-potential were measured by dynamic light scattering, and the presence as well as the percentage of coating molecules on the MNP surface were analyzed by infrared spectroscopy and thermogravimetric analyses, respectively. MNP morphology was studied by transmission electronic microscopy (TEM) and their magnetic properties were analyzed in a vibrating sample magnetometer.

### Cell Culture

The human NK-92MI cell line (kindly provided by Dr. A. Pérez-Martínez, IdiPaz, Madrid, Spain) was cultured in RPMI1640 supplemented with 5% FBS, 5% human serum (Sigma-Aldrich), 2 mM L-glutamine, 100 U/ml penicillin/streptomycin (P/S), 1 mM sodium pyruvate, 50 μM 2-mercaptoethanol, 10 mM HEPES, 1X non-essential amino acids (complete RPMI medium), and 50–100 U/ml recombinant human IL-2 (Peprotech) when required, under standard culture conditions (37°C, 5% CO_2_, 90% relative humidity). The murine tumor cell lines YAC-1 (ATCC: TIB-160) and RMA/S (courtesy of Dr. B. Chambers, Karolinska Institute, Sweden) as well as the human tumor cell line K562 (provided by Dr. A. Pérez-Martínez, IdiPaz, Spain) were cultured in RPMI1640 with 10% FBS, 2 mM L-glutamine, and 100 U/ml P/S. The murine endothelial cell line SVEC4-10 (ATCC: CRL-2181) was cultured in DMEM with 10% FBS, 2 mM L-glutamine, 1 mM sodium pyruvate, and 100 U/ml P/S. Cells were cultured under standard conditions at all times.

Murine NK cells were purified from the spleens of 12–20 weeks old C57BL/6 mice (Jackson Laboratories). These spleens were processed to obtain the cell suspension following erythrocyte lysis. We then used the positive selection Anti-NKp46 Microbead Kit (mouse) (Miltenyi Biotec) to isolate murine NK cells, following the manufacturer's instructions. Once isolated, they were cultured in 96-well U-bottom culture plates using the complete RPMI medium supplemented with murine recombinant IL-2 (1,000 U/ml, Peprotech) and expanded for 7 days. The percentage of NK cells (CD3^−^NKp46^+^) was checked by flow cytometry at day 0 and day 7, obtaining a purity of around 90–95% after expansion. At this point they were used in the corresponding experiments.

### Mice

C57BL/6 mice were purchased from Jackson Laboratories, housed in the CNB animal facility, and handled according to the recommendations of the CNB-CSIC institutional ethics committee. Procedures involving animals were approved by the CSIC ethics committee for animal experimentation and by the Division of Animal Protection of the regional government of Madrid in compliance with national and European Union legislation.

### Cell Viability

Cell viability was studied by Alamar Blue assay (Invitrogen) and FITC-annexin V/propidium iodide staining. In the former, either the murine NK cells expanded *in vitro* in the presence of IL-2 or NK-92MI cells were incubated with different MNP concentrations for 24 h, after which Alamar Blue was added and then cultured for an additional 4 h, at which point fluorescence was measured. For the second assay, cells were processed using the Annexin V-PI apoptosis assay kit following the manufacturer's protocol (Life Technologies) and analyzed by flow cytometry.

### MNP Uptake and Release

To quantify MNP uptake by inductively coupled plasma-optical emission spectrometry (ICP-OES), murine NK cells expanded *in vitro* in the presence of IL-2 or the human NK-92MI cell line (10^7^ cells/ml) were treated with MNPs (150 μg Fe/ml) for 2 h, after which they were washed to eliminate the excess of MNPs and digested at 90°C with HNO_3_ 63% and then with H_2_O_2_.

To evaluate the MNP release from cells, the human NK-92MI cell line (10^7^ cells/ml) were treated with MNPs (150 μg Fe/ml) for 2 h, after which they were washed to eliminate the excess of MNPs and cultured in 1 ml medium for several timepoints (24, 48 and 72 h). At these timepoints, the supernatant was collected, digested at 90°C with HNO_3_ 63% and then with H_2_O_2_ and the presence of iron was measured by ICP-OES.

### Microscopy Analysis

For confocal microscopy, we stained the *in vitro*-expanded murine NK cells or the human NK-92MI cell line after MNP treatment with LysoTracker Red DND-99 (Life Technologies), Alexa Fluor 647-wheat-germ agglutinin (Life Technologies), and DAPI. Finally, the samples were mounted in Fluoromount-G (Southern Biotec), and images were acquired using a confocal multispectral Leica TCS SP5 system with a 63 × /1.4 NA oil-immersion objective. To visualize the MNPs, dark-field microscopy was used. For TEM, expanded murine NK cells or the human NK-92MI cell line, some treated with MNPs and some without treatment, were fixed and processed by the Transmission Electron Microscopy Service at the National Center for Biotechnology (CNB-CSIC, Madrid, Spain). Images were acquired with a JEOL JEM 1,011 transmission electron microscope at various magnifications.

### Flow Cytometry and Proliferation Assays

The following primary anti-mouse antibodies were used: anti-NK1.1 (PK136), -CD69 (H1.2F3), -CD62L (Mel-14), -Ly49A+D (12A8), and -Ly49D (4E5) from Pharmingen, -CD3 (17A2, eBioscience), -NKp46 (29A.1, Biolegend), -NKG2D (CX5, Invitrogen), -CD27 (LG.3A10, BD), -CD11b (M1/70, Beckman C.), and -Ly49F (HBF-719, Coulter). The following primary anti-human antibodies were used: anti-CD56 (N901 (NKH-1)), -CD95 (7C11), -HLA-ABC (B9.12.1), -CD27 (1A4CD27), -CD11b (BEAR-1) and -CD11a (25.3.1) from Immunotech, -NKp46 (9E2), -KLRG1 (SA231A2), and -DNAM-1 (11A8) from Biolegend, -CD16 (3G8) and -CD25 (B1.49.9) from Beckman C., -CD69 (FN50, eBioscience), -NKG2D (1D11, BD), and -IFN-γ (B27, Pharmingen). Data were acquired on a FC500 flow cytometer and analyzed with FlowJo software.

For intracellular protein labeling, cells were permeabilized by washing them with a 0.5% saponin in PBS staining buffer. Then, they were subsequently labeled with the corresponding antibodies, which had been diluted in this solution, for 30 min at 4°C. The cells were then washed two times with PBS and resuspended in PBS for analysis by flow cytometry.

For proliferation assays, NK-92MI cells were labeled with the CellTrace CFSE cell proliferation kit (Thermofisher) as described in Rojas et al. (54). Cell were loaded with 150 μg/mL APS-MNP (or left untreated as control) and proliferation evaluated after 0, 48 and 96 h.

### Conjugation Assays

Either murine NK cells expanded *in vitro* in the presence of IL-2 or the human NK-92MI cell line, treated with APS-MNPs or not, as well as the corresponding cellular targets (murine RMA/S and YAC-1 cell lines for murine NK cells and the human K562 cell line for NK-92MI cells) were stained using the fluorescent labeling kits, consisting of dyes for red (PKH26) or green (PKH67) (both from Sigma-Aldrich), alternating labeling between the effector and target cells in the different experiments. Murine or human NK cells and their corresponding target cells were co-incubated for varying periods of time at a 1:1 ratio under standard incubation conditions, after which they were fixed in 1% PFA and analyzed by flow cytometry. Each procedure was performed in duplicate. The events positive for both colors were considered to be conjugated between the NK cell and its target, and the percentage of conjugated NK cells was calculated as follows: (% conjugated NK cells / % total NK cells) × 100%.

### Degranulation Assays

We incubated 2 × 10^5^ murine NK cells expanded *in vitro* in the presence of IL-2 or the human NK-92MI cell line, treated with APS-MNPs or not, with different stimuli and in the presence of 10 μg/ml of monensin (Sigma-Aldrich) and 5 μl of the CD107a antibody conjugated with FITC (or its corresponding control). After 4–5 h of incubation under standard conditions, CD3 and NKp46 (murine) or CD56 (human) markers were labeled for cytometry. The percentage of CD3^−^NKp46^+^CD107a^+^ cells (for murine NK cells) or CD56^+^CD107a^+^ cells (for human NK-92MI cells) was analyzed by flow cytometry.

A variety of stimuli were used in these assays. Murine NK cells expanded *in vitro* in the presence of IL-2 were seeded in 96-well plates previously coated with the antibody anti-mouse NKG2D (10 μg/ml; A10, eBioscience), or -mouse NKp46 (5 μg/ml; 29A1.4, Biolegend), or -mouse NK1.1 (10 μg/ml; PK136, Biolegend), or the appropriate controls. NK-92MI cells were co-cultured with K562 cells at a 1:2 ratio (NK-92MI cells: K562 cells) or seeded in 96-well plates previously coated with the anti-human NKG2D antibody (10 μg/ml; 1D11, Pharmingen). Besides, brefeldin A or monensin were also added during the stimulation to inhibit protein transport and to detect intracellular CD107a marker.

### Cytolytic Activity Measurements

Expanded murine NK cells or the human NK-92MI cell line, treated with APS-MNPs or not, were co-incubated with their corresponding targets (murine RMA/S and YAC-1 cell lines for murine NK cells and the human K562 cell line for NK-92MI cells), which had been previously stained with the PKH67 Green fluorescent cell-linker kit (Sigma-Aldrich) at different ratios in a total volume of 200 μl for 4 h under standard incubation conditions and in 96-well U-bottom plates. Each procedure was performed in duplicate. The reaction was stopped by adding 250 μl of cold PBS staining, and the plates were placed in ice. Fifteen microliter of PI were added to each well just before flow cytometry analysis. The percentage of specific lysis was calculated for each target cell as follows: % lysis = (% dead target cells—% spontaneous death) / (100—% spontaneous death) × 100%. The percentage of spontaneous death was calculated in the absence of effector cells.

### Intracellular Staining of IFN-γ

NK-92MI cells treated with APS-MNPs or not were exposed to different stimuli to determine their IFN-γ production capacity by analyzing intracellular IFN-γ levels using flow cytometry. To do this, 2 × 10^5^ effector cells were incubated with certain stimuli or specific targets in the presence of brefeldin A (1X, Biolegend). After 4–5 h of incubation under standard conditions, they were labeled for CD3 and NKp46, at which point intracellular staining was carried out. The percentage of CD56^+^IFN-γ^+^ cells was analyzed by flow cytometry.

The different stimuli used in these assays were the following: co-incubation with K562 cells at a 1:2 ratio (NK-92MI cells: K562 cells) or 25 ng/ml PMA and 1 μg/ml ionomycin, both from Sigma-Aldrich. In addition, brefeldin A or monensin were also added during the stimulation to inhibit protein transport and to detect intracellular IFN-γ.

### ELISA (IFN-γ)

The supernatants obtained during the degranulation experiment performed with the *in vitro-*expanded murine NK cells were collected and the production of IFN-γ (produced by the NK cells) was analyzed by the commercial ELISA kit BD OptEIA Mouse IFN-γ ELISA Set following the manufacturer's instructions.

### Adhesion and Transmigration Capacity Evaluation

We seeded 3–4 × 10^4^ murine endothelial cells (SVEC4-10 cells) on coverslips with a 12-mm diameter, placing these in a 24-well culture plate. These were then cultured under standard conditions until the monolayer was formed, after which the monolayer was activated with murine TNFα (250 U/ml, Peprotech) for 6 h. In addition, murine NK cells expanded *in vitro* in the presence of IL-2 were labeled with the CellTrace CFSE probe (2.5 μM, ThermoFisher Scientific) following the manufacturer's instructions. Once labeled, they were incubated with different concentrations of APS-MNPs for 2 h under standard conditions, after which 5 × 10^4^ cells were seeded on the activated endothelium monolayer and incubated for 1 h. Subsequently, the cells over the monolayer were washed with PBS, fixed with 4% PFA for 20 min, and the actin filaments were stained with phalloidin-TRITC (1: 500, Sigma-Aldrich) for 45 min at RT in the dark. Finally, the cells were washed with PBS and mounted on slides with Fluoromount-G for observation by confocal microscopy. The images were acquired with the Leica TCS SP5 confocal microscope, using the 10 × and 20 × lenses, sweeping the entire monolayer in 1-μm slices. The images obtained were analyzed by ImageJ software.

### Flow Chamber Assays

*In vitro* magnetic retention assays were carried out under flow conditions in a modified channel slide (μ-Slide I Luer, 0.4-mm height, ibidi) using a two-magnet system as previously described (43). The neodymium iron boron (NdFeB) permanent magnets (Supermagnete) used in this study were the following: an 5 × 14-mm magnet with 1.35 T of remanent magnetization (Br) (magnet A) and an 8 × 6-mm magnet with 1.45 T of remanent magnetization (Br) (magnet B). Briefly, either murine NK cells expanded *in vitro* in the presence of IL-2 or the human NK-92MI cell line (10^7^ cells/ml) were treated with MNPs (150 μg Fe/ml) or not for 2 h in standard conditions, and then washed and stained with calcein-AM. An Olympus Inverted Microscope (model IX71) coupled to a Cell^∧^R imaging station under standard culture conditions was used in this assay. Shear stress was fixed at 0.5 dyne/cm^2^ and events were recorded every 1 s. After 60 s, the two-magnet system was applied for the next 2 min. Imaris software (Bitplane) was used to analyse displacement along the Y-axes (in the direction of the magnetic field).

### Transwell Migration Assay

MNP-treated and -untreated murine NK cells expanded *in vitro* in the presence of IL-2 or the human NK-92MI cell line were differentially labeled with PKH26 Red or PKH67 Green fluorescent cell-linker kits (Sigma-Aldrich) and mixed at a 1:1 ratio, after which 5 × 10^5^ cells were seeded in 0.1 ml of the appropriate medium in a transwell insert (Corning, 5-μm pore). The chemotactic gradient was created by adding the recombinant murine fractalkine (CX3CL1, aa25-105) (100 ng/ml, R&D Systems) for murine NK cells or the recombinant human CXCL12 (100 ng/ml, Peprotech) for human NK-92MI to the lower chamber. The cells migrated for 16 h, after which cells from lower chambers were counted by flow cytometry. Cell migration was quantified and normalized for loading into an input well. In some cases, the magnet B was placed below the well.

### Statistical Analyses

The graphs and statistical analyses were made with Prism 5.0 (GraphPad) software. All analyses were performed using one-way ANOVA and Tukey's multiple comparisons *post-hoc* test. ^*^p < 0.05, ^**^p < 0.01, ^***^p < 0.001, ^****^p < 0.0001.

## Results

### Preliminary Assessment of MNP Interaction With the Human NK-92MI Cell Line

Several coated MNPs were synthesized and characterized to determine, in an initial study, which was the best to use in this approach. These MNPs had an iron-oxide core of 12.5 nm and had different surface charges dependending on their coating. By doing this, we obtained positively charged (APS-MNPs), negatively charged (DMSA-MNPs), and non-charged MNPs (DEXT-MNPs). Their complete physico-chemical characterization has been previously described [Table S1; (43)].

When screening the MNPs to determine which was the most appropriate for this cell type, we first analyzed the interaction between the human NK cell line, NK-92MI, and the different MNPs, as well as the toxicity they could cause to this cell line.

**Figure 1:**
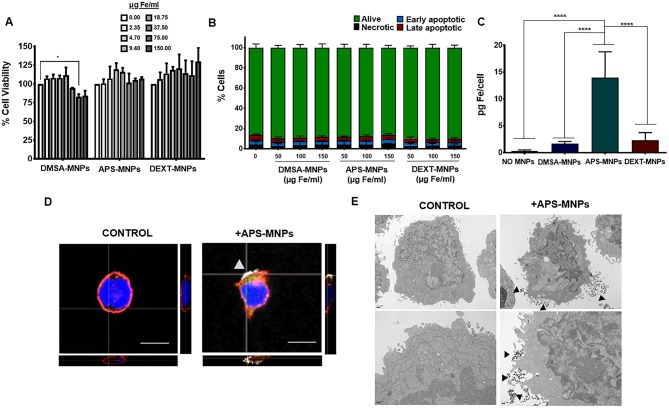
Evaluation of MNP toxicity, quantification of MNP uptake, and its subcellular location in NK-92MI cells. **(A)** Viability of NK-92MI cells after treatment with MNPs, as measured by the AlamarBlue fluorometric test. **(B)** Analysis by flow cytometry of the percentage of apoptotic or necrotic human NK-92MI cells after incubation with MNPs by Annexin V/PI staining. (Alive: Annexin V^−^/PI^−^; early apoptotic: Annexin V^+^/PI^−^; late apoptotic: Annexin V^+^/PI^+^ and necrotic: Annexin V^−^/PI^+^). **(C)** Quantification of the iron associated with the NK-92MI cells after incubation with the different MNPs, through ICP-OES. The results shown (mean ± SD) are representative of three independent experiments, *p < 0.05, ****p < 0.0001. **(D)** Representative images of the NK-92MI cells after treatment with APS-MNPs acquired by confocal microscopy [cell membrane (red), NPMs (gray), and nucleus (blue)] (scale = 10 μm). The orthogonal projections were composed using ImageJ software. **(E)** Representative images obtained by TEM depicting NK-92MI cells after treatment with MNPs. The upper panels offer an overall view of the cell, while the lower panels show cell regions in greater detail to better illustrate the interactions between MNPs and the cell membrane.

To study MNP toxicity, we analyzed cell survival after MNP incubation using two different assays. In general, MNP treatment did not affect NK-92MI cell viability, although a certain decrease in the viability of these cells was found after they were treated with high doses of DMSA-MNPs (Figure 1A). Furthermore, analysis of apoptosis/necrosis by flow cytometry showed no significant changes when the cells were incubated with the different MNPs (Figure 1B). We also demonstrated that APS-MNP treatment at high dose (150 μg/ mL) did not affect NK-92MI proliferation at 48 or 96 h (Figure S1).

Secondly, the amount of MNPs associated with this immune-cell type was further assessed. ICP-OES measurements revealed that APS-MNPs led to higher iron detection (14.0 ± 4.8 pg Fe/cell in APS-MNP-treated NK cells vs. 1.7 ± 0.4 pg Fe/cell and 3.5 ± 0.9 pg Fe/cell when treated with DMSA-MNPs and DEXT-MNPs, respectively) (Figure 1C). Taking these results into account, we selected the MNPs coated with APS (APS-MNPs) to continue with the study. We also evaluated the release of APS-MNP by ICP-OES measurements in the culture supernatant of NK-92MI cells after 24, 48, and 72 h. The amount of iron found in the medium at the different timepoints (12.5 ± 3.8 μg Fe/ml at 24 h, 14.0 ± 6.7 μg Fe/ml at 48 h and 8.1 ± 1.5 μg Fe/ml at 72 h) were low compared to the amount used to treat the cells (150 μg Fe/ml), indicating that only a small fraction of the iron is being slowly released from the surface of MNP-treated cells.

Furthermore, the subcellular location of these APS-MNPs was determined by means of different microscopy approaches. Confocal microscopy showed that these APS-MNPs remained associated with the plasma membrane (Figure 1D), and a more detailed study through TEM revealed close interaction between the cell membrane and the MNPs (Figure 1E).

### Evaluation of the Functionality of the Human NK-92MI Cell Line After MNP Treatment and Magnetic Retention of These Cells *in vitro*

**Figure 2:**
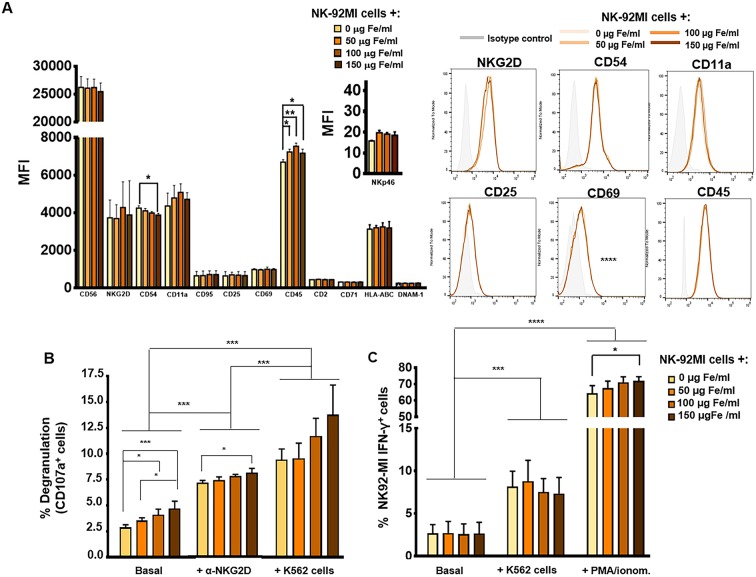
Phenotypic and functional analysis of NK-92MI cells after their association with MNPs. **(A)** Quantification (MFI, mean fluorescence intensity) of the expression of relevant cell-surface markers in NK-92MI cells, after incubation with MNPs and representative histograms. **(B)** Degranulation capacity of NK-92MI cells, after being treated with different concentrations of MNPs and after exposure to a range of stimuli. **(C)** Quantification of the percentage of IFN-γ^+^ NK-92MI cells after MNP treatment and exposure to a number of stimuli. The data (mean ± SD) are representative of three independent experiments, *p < 0.05, **p < 0.01, ***p < 0.001, ****p < 0.0001.

NK cells are characterized by the presence of inhibitory and activating receptors on their cell surface, which are essential to carry out their main function (55, 56). In addition, other cell-surface molecules such as the activation markers CD69 and CD25, as well as adhesion molecules such as CD54 (ICAM-1), CD11a, or CD62L also play an important role in the activity of these cells. For all these reasons, the expression of these receptors and molecules is important to the functionality of NK cells, making it necessary to analyse their expression after MNP treatment. To do this, the expression of some of these relevant surface molecules was analyzed by flow cytometry after incubation with various concentrations of the selected MNPs (APS-MNPs). As seen in Figure 2A, no significant differences were observed in the expression of the different surface molecules analyzed in the NK-92MI cells (CD56, NKG2D, CD11a, CD95, CD25, CD69, CD45, DNAM-1, or CD2) with the exception of a slight decrease in the expression of the adhesion molecule CD54 at high doses of MNPs (4,246 ± 120 of MFI in the absence of APS-MNPs vs. 3,812 ± 961 of MFI in the presence of 150 μg Fe/ml of APS-MNPs) as well as an increase in CD45, which is related to the maturation of lymphoid cells (6,687 ± 136 of MFI in the absence of MNPs vs. 7,232 ± 142, 7,531 ± 166 and 7,163 ± 214 of MFI in the presence of increasing concentrations of MNPs) [Figure 2A; (57)]. A slight increase, although not statistically significant, in NKp46 expression was also detected in APS-MNP loaded NK-92MI cells. As iron presence can alter MHC class I (58) and transferrin receptor (CD71) expression, we also assessed the expression of these markers in NK-92MI cells (Figure 2A). APS-MNP loading did not alter the expression of HLA-A,B,C, or CD71 in these cells. NK-92MI cells were negative for CD11b, CD27, and KLRG-1 expression and APS-MNP exposure did not induce the expression of these markers (Figure S2). It thus appear that APS-MNP treatment has minimal effects on NK-92MI surface markers.

Furthermore, as mentioned above, the membrane protein associated with lysosome CD107a is increased on the surface of NK cells after degranulation and activation by certain stimuli with a capability of inducing a cytolytic response. The expression of CD107a correlates with lysis mediated by NK cells, so it is used as a marker to determine whether these cells develop defects in their cytolytic function (59). After stimulation of NK cells with the K562 target cell line, or through cross-linking with a ligand for NK cell-activating receptor such as α-human NKG2D, we did not observe, in general, significant differences in CD107a expression when using increasing concentrations of APS-MNPs, even though basal or non-specific degranulation was increased (2.9 ± 0.3% in the absence of MNPs vs. 3.6 ± 0.3%, 4.1 ± 0.5%, and 4.7 ± 0.7% in the presence of increasing concentrations of MNPs) (Figure 2B). The results also showed a slight increase in the degranulation obtained after stimulation with the α-human NKG2D when treated with 150 μg Fe/ml of APS-MNPs (7.2 ± 0.2% in the absence of MNPs vs. 8.1 ± 0.3% with 150 μg Fe/ml of APS-MNPs) (Figure 2B).

Another important aspect in the functionality of NK cells is the rapid production of various pro-inflammatory cytokines, one of the most important of which is IFN-γ (8). Therefore, their capacity to produce IFN-γ after being co-cultured with the K562 target cell line or in the presence of PMA/ionomycin was further assessed by flow cytometry. The results showed that this capacity remains intact after MNP treatment. However, the percentage of NK-92MI IFN-γ^+^ cells was slightly increased after treatment at high doses of MNPs and when exposed to PMA/ionomycin [64.5 ± 4.6% in the absence of MNPs and 72.1 ± 2.4% at the highest dose of MNPs (150 μg Fe/ml)] (Figure 2C).

**Figure 3:**
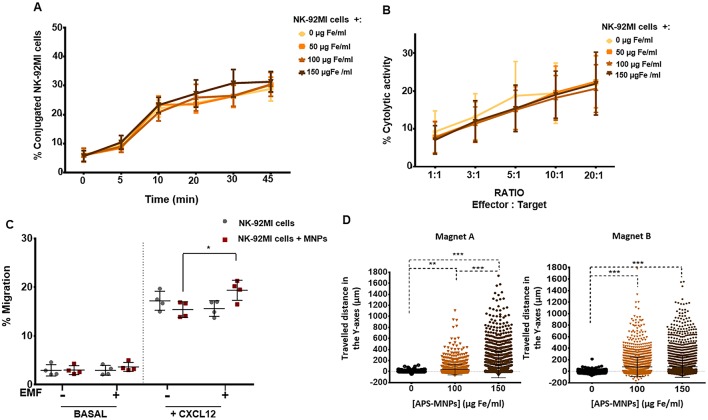
Functional assessment of NK-92MI cells after their association with MNPs and *in vitro* magnetic retention. **(A)** Conjugation kinetics of NK-92MI cells, after being treated with MNPs, with the K562 cell line, co-incubated in a 1:2 ratio. **(B)** Cytolytic activity of NK-92MI cells, after treatment with different concentrations of MNPs, against the K562 cell line, co-incubated in different ratios for 4 h. **(C)** Migratory capacity of NK-92MI cells after treatment with MNPs in response to a specific chemotactic gradient and in the presence or absence of an EMF in the same direction. The results were normalized against a control well (in the absence of a transwell assay). **(D)** Displacement of NK-92MI cells in the direction of the magnetic gradient (Y axis) after being treated or not with MNPs and exposed to different EMFs. Cell displacement was quantified by analyzing at least 100 cells per video using Imaris software. The results shown (mean ± SD) are representative of three or four independent experiments, *p < 0.05, **p < 0.01, ***p < 0.001.

In addition, we proceeded to determine whether the NK-92MI cells evidenced defects in their conjugation capacities with different target cells following MNP treatment. Conjugation with target cells is of great relevance, as physical interaction among these cells is a key step with which NK cells exert their cytolytic activity (60). No significant differences were detected in the conjugation kinetics obtained between the human NK cells left free of APS-MNPs or associated with different concentrations of APS-MNPs and the K562 human cell line (Figure 3A). Further, no differences or defects in the conjugation of these cells were found, even in the presence of APS-MNPs, both after short times (2–5 min), when they always remained around 2–4%, and at longer times (45 min), around 30–40% in both cases.

Furthermore, we evaluated whether the association between the NK-92MI cells and APS-MNPs affected their direct cytolytic capacity against target cells. To do this, the percentage of lysed target cells at different effector-target cell ratios was analyzed. No significant differences were observed in the different, increasing concentrations of APS-MNPs assessed (9.3 ± 5.5% lysis in the absence of MNPs vs. 7.1 ± 3.7% lysis in the presence of 150 μg Fe/ml of APS-MNPs at lower ratios in co-culture with K562 cells and 21.8 ± 7.4% lysis vs. 22.0 ± 8.2% lysis, respectively, at higher ratios) (Figure 3B).

After analyzing the activation and cytolytic activity of APS-MNP–loaded NK-92MI cells, we proceeded to evaluate the capacity of these cells to respond to a biological factor such as a chemotactic gradient. NK cells respond to various chemokines, which directly affects their location both during their development and maturation and during an immune response (61). Transwell assays showed that the presence of APS-MNPs in the NK-92MI cells affected the cell migration in a mild but non-significant way in response to a chemotactic gradient (17.2 ± 1.9% migration in the absence of APS-MNPs vs. 15.4 ± 1.6% migration in the presence of APS-MNPs) (Figure 3C). In addition, the application of an EMF in the same direction as in the chemotactic gradient produced an increase in the migration of the cells associated with the MNPs (15.4 ± 1.6 vs. 19.4 ± 2.1% migration in the presence of MNPs and in absence or the presence of an EMF, respectively) (Figure 3C).

As NK cells are continuously circulating between the blood and different tissues, we proceeded to study whether it was possible to magnetically retain these APS-MNP-loaded NK-92MI cells in a dynamic flow system. We assessed different concentrations of MNPs as well as different magnetic forces, measuring the displacement of these cells toward the magnetic gradient to which they were exposed. This retention increased with greater numbers of APS-MNPs associated with the cells (56 ± 97 vs. 124 ± 236 μm when treated with 100 and 150 μg Fe/ml, respectively, and using the magnet A) and with higher strength of the magnetic gradient (Figure 3D).

### Application to a Primary NK Cell Model: Murine NK Cells Expanded *in vitro* in the Presence of IL-2

Once the interaction between the MNPs and the human NK-92MI cell line had been carefully evaluated, we moved to primary murine NK cells, which were isolated from the spleen of mice and expanded *in vitro* in the presence of IL-2 for 7 days. The purity of these primary cultures was always checked at day 0, after isolation, and at day 7, after expansion with IL-2 (Figure S3).

**Figure 4:**
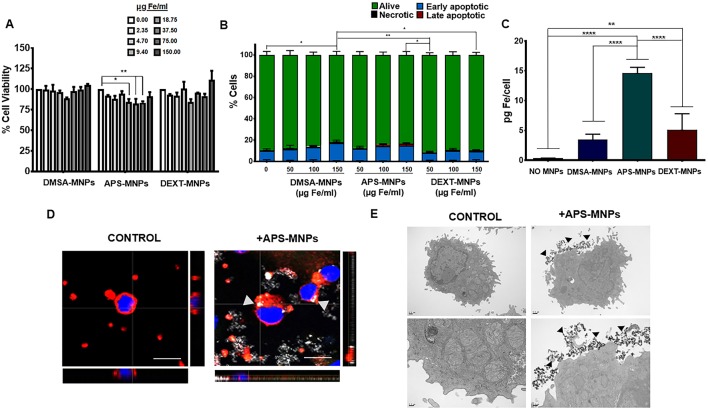
Evaluation of MNP toxicity, quantification of MNP uptake and determination of subcellular location in murine NK cells. **(A)** Cell viability of murine NK cells after treatment with MNPs, measured by the AlamarBlue fluorometric test. **(B)** Analysis by flow cytometry of the percentage of apoptotic or necrotic murine NK cells after incubation with MNPs by Annexin V/PI staining. (Alive: Annexin V^−^/PI^−^; early apoptotic: Annexin V^+^/PI^−^; late apoptotic: Annexin V^+^/PI^+^ and necrotic: Annexin V^−^/PI^+^). **(C)** Quantification of the iron associated with the murine NK cells after incubation with the different MNPs, through ICP-OES. The results shown (mean ± SD) are representative of three independent experiments, *p < 0.05, **p < 0.01, ***p < 0.001, ****p < 0.0001. **(D)** Representative images of the murine NK cells after treatment with APS-MNPs acquired by confocal microscopy [cell membrane (red), NPMs (gray), and nucleus (blue)] (scale = 10 μm). The orthogonal projections were composed using ImageJ software. **(E)** Representative images obtained by TEM of murine NK cells after treatment with MNPs. The upper panels offer an overall view of the cell, while the lower panels depict cellular regions in greater detail to better illustrate the interactions between MNPs and the cell membrane.

First, we confirmed that the different MNPs used in this study did not cause significant toxicity over these primary NK cells (Figures 4A,B) by applying the same methods used previously to assess viability of the human NK cell line. However, a slight decrease in cell viability in the presence of certain concentrations of APS-MNPs was observed (Figure 4A). Similarly, the analysis of apoptosis/necrosis by flow cytometry showed no significant changes when the cells were incubated with the different MNPs, but a slight increase in early apoptosis was seen in the presence of DMSA-MNPs or APS-MNPs (8.8 ± 2.1% in the absence of MNPs vs. 16.5 ± 2.8 and 14.5 ± 2.6% after the highest doses of DMSA- and APS-MNPs) (Figure 4B).

We also verified that the APS-MNPs were the MNPs that associated the most with these cells, around 14–15 pg Fe per cell (Figure 4C). This value was very similar to the one previously found in the human NK cell line. In addition, we visualized the association between APS-MNPs and the primary murine NK cells by confocal microscopy and TEM. Both imaging techniques illustrated the interaction between the APS-MNPs and the cell surface of these murine NK cells, as in the human NK-92MI cell line (Figures 4D,E).

**Figure 5:**
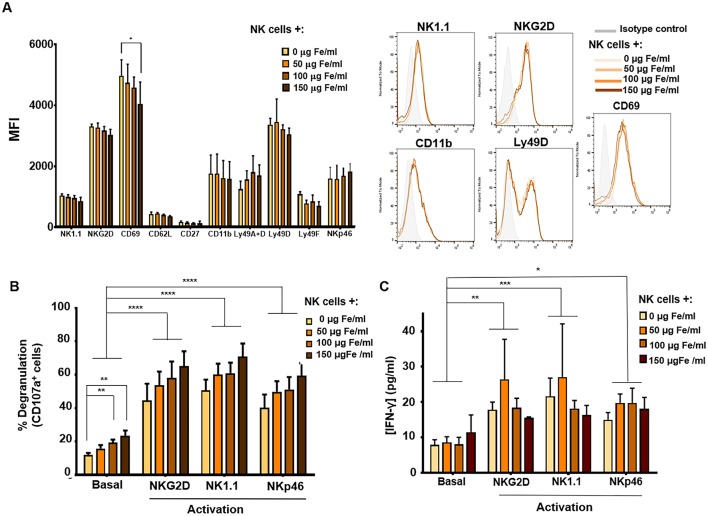
Phenotypic and functional analysis of murine NK cells after their association with MNPs. **(A)** Quantification (MFI, mean fluorescence intensity) of the expression of relevant cell-surface markers in murine NK cells after incubation with MNPs and representative histograms. **(B)** Degranulation capacity of murine NK cells after treatment with different concentrations of MNPs and after exposure to a variety of stimuli. **(C)** Quantification of IFN-γ production by murine NK cells after MNP treatment and exposure to a range of stimuli, measured by ELISA assay. The data (mean ± SD) are representative of three independent experiments, *p < 0.05, **p < 0.01, ***p < 0.001, ****p < 0.0001.

We then continued by assessing the effect APS-MNPs could have on the phenotype and the expression of several surface molecules of these primary murine NK cells by flow cytometry. No significant differences were observed in the expression of most of the different markers analyzed in IL-2 expanded murine NK cells after MNP treatment (NK1.1, NKG2D, CD62L, CD27, CD11b, Ly49A+D, Ly49D, Ly49F, or NKp46), though a slight decrease in the CD69 activation marker was observed after treatment with high concentrations of APS-MNPs (4,974 ± 518 vs. 4,051 ± 713 of MFI) (Figure 5A). Therefore, APS-MNPs did not significantly influence the murine NK phenotype.

The degranulation capacity of these primary murine NK cells as well as their ability to produce IFN-γ when associated with APS-MNPs was further evaluated. After stimulation through cross-linking with NK cell activating receptor ligands (such as NKG2D, NK1.1, and NKp46) (62, 63), we determined the presence of the degranulation marker in these NK cells when associated with increasing concentrations of APS-MNPs. No significant differences were observed in the degranulation of these cells in the presence of the MNPs after the different stimuli, even though basal or non-specific degranulation was significantly increased (12.0 ± 2.5% in the absence of MNPs vs. 19.3 ± 4.1 and 23.5 ± 7.0% in the presence of the highest concentrations of APS-MNPs) (Figure 5B).

In addition, we analyzed the production of IFN-γ by murine NK cells expanded in the presence of IL-2 in the presence of different concentrations of APS-MNPs and after exposure to various stimuli. This factor was analyzed after activation of the murine NK cells by cross-linking with ligands of different activating receptors (NKG2D, NK1.1, and NKp46) by means of an ELISA assay (62, 63). No significant differences were observed in the production of IFN-γ after incubation with the APS-MNPs (Figure 5C).

**Figure 6:**
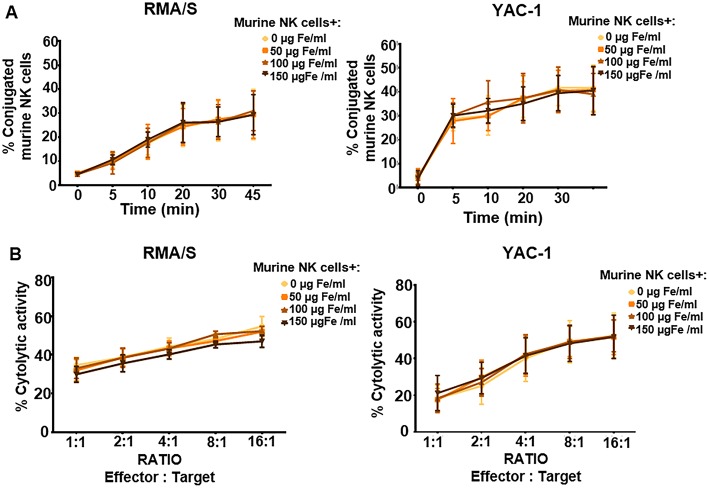
Functional assessment of murine NK cells after their association with MNPs. **(A)** Conjugation kinetics of NK-92MI cells after being treated with MNPs, with RMA/S (left panel) and YAC-1 (right panel) cell lines co-incubated in a 1:2 ratio. **(B)** Cytolytic activity of murine NK cells, after being treated with different concentrations of MNPs, against RMA/S (left panel) and YAC-1 (right panel) cell lines co-incubated at different ratios for 4 h. The data (mean ± SD) are representative of three or four independent experiments.

We then assessed in detail the conjugation capacity and cytotoxicity when treated with increasing concentrations of APS-MNPs and co-cultured with the YAC-1 and RMA-S murine cell lines, which are susceptible to being lysed by the murine NK cells. No significant differences in conjugation were detected between the primary murine NK cells that remained free from APS-MNPs and when these cells were associated with different concentrations of APS-MNPs (Figure 6A), and no difference was found in their cytolytic activity (Figure 6B).

**Figure 7:**
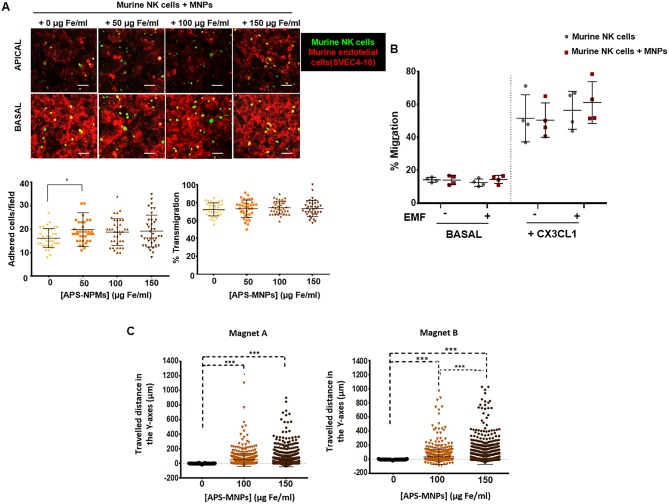
Transmigration and *in vitro* retention of MNP-loaded murine NK cells through the application of an EMF in flow chambers and analysis of their chemotactic response. **(A)** Representative images of murine NK cells, treated with NPMs or not, migrating over a monolayer of murine endothelial cells (SVEC-4 cell line), acquired through confocal microscopy. The upper panel represents the apical part of the monolayer, while the lower panel represents the basal part [murine NK cells (green), cytoskeleton (red)]. Scale: 100 μm. Quantification of the adhesion (left panel) and transmigration (right panel) exhibited by murine NK cells in the presence of increasing concentrations of MNPs through murine endothelial cells. The results shown (mean ± SD) are representative of three independent experiments, analyzing at least 50 fields per condition, *p < 0.05, ***p < 0.001. **(B)** Migratory capacity of murine NK cells after treatment with MNPs in response to a specific chemotactic gradient and in the presence or absence of an EMF in the same direction. The results of this test were normalized against a control well (in the absence of transwell assay). **(C)** Displacement of murine NK cells in the direction of the magnetic gradient (Y axis) after being treated with MNPs or not and exposed to different EMFs. Cell displacement was quantified by analyzing at least 100 cells per video using Imaris software. The results shown (mean ± SD) are representative of three or four independent experiments, *p < 0.05, ***p < 0.001.

The process of lymphocyte recruitment, which comprises a complex cascade of different events starting with the adhesion of the cells to the endothelium and through to the transendothelial migration (64), plays an essential role during the immune response. We therefore evaluated whether the presence of APS-MNPs on the surface of the murine NK cells affected their adhesion to an endothelium, as well as the ability of these cells to transmigrate. No significant differences were observed in adhesion capacity (Figure 7A) or ability to transmigrate (around 73% transmigration at all times) (Figure 7A). However, a slight increase in adhesion capacity was observed in the presence of APS-MNPs (16 ± 4 cells adhered/field in the absence of MNPs vs. 20 ± 7 cells adhered/field in the presence of the lowest dose of MNPs) (Figure 7A), possibly due to the presence of the MNPs on the surface, which could interact with the endothelial cells.

Further analyses of the chemotactic response of the primary murine NK cells in the presence of APS-MNPs revealed that the presence of MNPs on the cell surface did not significantly affect the migration of these cells in response to a chemotactic gradient. However, the application of an EMF in the same direction as the chemotactic gradient produced a slight but not significant increase in the migration of the primary murine NK cells associated with the MNPs (50.4 ± 10.5 vs. 61.1 ± 12.7% migration in the presence of APS-MNPs and in the absence and presence of an EMF, respectively) (Figure 7B).

Similarly to NK-92MI cells (Figure 4D), the magnetic retention of APS-MNP-loaded primary murine NK cells in the presence of flow forces (Figure 7C) increased with greater APS-MNP concentrations as well as with the strength of the magnetic force.

## Discussion

NK cells are known to play an essential role in host immunity to cancer and are ideal candidates for use in the fight against this complex disease. Furthermore, NK cell-transfer therapy has proven to be an effective means of treating some adult hematological malignancies, thus reinforcing the idea that NK cells can be an important therapeutic tool for the treatment of other types of cancer. In fact, some therapies based on this concept are already being tested at the clinical level, although some with moderate results (32). As with other cell-based therapies, NK cell-transfer therapy has limitations, such as the dispersion of *in vivo*-administered NK cells, and strategies designed to solve this problem are being studied widely. One promising approach combines MNPs and EMFs to localize specific cell types in a region of interest. This approach has proven efficient when different types of cells are used in targeting, including NK cells. For example, previous studies showed how lymphoid cells such as T cells, when associated with MNPs, can be magnetically retained in the lymph nodes, which could be useful in modulating the immune response in the context of disease (43). This strategy can also be applied to other regions of interest, such as a tumor, increasing the antitumor immune response carried out by activated effector cells. To date, some studies have used this type of strategy to accumulate stem cells, mesenchymal cells, macrophages, or dendritic cells as part of tissue-regeneration therapies or in therapy for autoimmune disorders (29, 36–40). However, use of MNPs and EMFs to redirect lymphoid cells such as T or NK cells for the treatment of cancer is limited and has been scarcely investigated (29, 30). One of these studies (30), showed that NK92MI cells loaded with MNP by magnetic transfection can be targeted to the tumor site by an EMF, but the *in vitro* functional analysis of MNP-loaded NK92MI cells was limited to a cytotoxicity assay. As such, a more extensive evaluation of the effects that MNP loading could have on NK cell function as well as optimization of magnetic retention are required for these approaches to reach the clinic. In addition, primary NK cells are known to be highly resistant to transfection methods (41, 42), likely decreasing the efficacy of these approaches.

We have previously shown that APS-MNPs attached to the surface of primary murine T cells can be specifically targeted to lymph nodes by using an EMF (43). This prompted us in this study to examine the influence of MNPs on the functionality of NK cells so as to determine the extent to which specific magnetic retention of fully functional NK cells could be promoted. To this end, we first assessed a model with great clinical significance: NK-92MI, a human NK cell line that derives from the clinically approved NK-92 cell line (26, 48, 49) subsequently moving on to a model of primary murine NK cells.

It is commonly described that MNPs seldom cause significant toxicity in different immune-cell types, with over 80% cell viability associated with this treatment approach (65). There are studies, however, that evidence the toxicity of some MNPs in lymphoid cells, primarily when high doses, certain coatings, and long exposure times are used (66–68). The MNPs employed here did not have a substantial toxic effect on the different models of NK cells, and as described previously, cell viability remained over 75–80% at all times. Primary murine NK cells displayed a slightly greater decrease in cell viability in some incubation conditions, probably due to higher susceptibility to apoptosis and senescence after a 7-days cytokine expansion.

The presence of the MNPs on the surface of the murine and human NK cells as observed by different types of microscopy correlated with previous studies using these MNPs and other types of lymphoid cells such as murine and human T cells (43). In this earlier study, the close association between the MNPs and the membrane of T cells did not significantly alter the main functional aspects of these cells, and this feature must be further evaluated in these NK cell models.

To date, only a few detailed studies have described the effect of MNPs on the functionality of cytotoxic lymphoid cells such as T and NK cells. Most analyse certain functional aspects of these cells after their association with MNPs in order to track these aspects by means of magnetic resonance imaging. Concerning T cells, some reports show that the proliferation of these cells in response to specific antigens, as well as their cytotoxic capacity against certain cell targets or the production of certain cytokines, do not seem to be affected by MNPs *in vitro* (69–72). There are also studies that show how effector T cells maintain their cytotoxic and antitumor activity *in vivo*, and these cells are able to migrate to and infiltrate the tumor with no apparent difficulty (73, 74). However, certain defects in the cytolytic capacity and cytokine production of these cells have been described, but only in the presence of very high doses of MNPs (> 500 μg Fe/ml) (67). Taken together, the studies described above seem to indicate that MNPs could be associated with effector T cells, possibly causing them to preserve their main functions.

Regarding NK cells, a small number of studies have evaluated the influence that MNPs can have on human NK cell lines such as NK-92 or KHYG-1, but none have explored this influence on primary human or murine NK cells. Existing studies show that NK cell lines preserve their cytolytic capacity *in vitro* and are able to migrate and infiltrate the tumor *in vivo* in much same way as when these cells are not associated with MNPs (30, 75–79). Therefore, these studies also seem to indicate that NK cells could associate with MNPs without impairment to their main functions. However, a more exhaustive analysis of these and other functional aspects of these cells, as well as of primary NK cells, is essential, and should test the degree to which these functions remain intact after association with MNPs, and in particular, with the particular MNPs used in this work (APS-MNPs).

In this study, we first analyzed the phenotype, showing that the association of the NK cells with APS-MNPs did not produce significant differences in the levels of expression of important surface markers such as adhesion molecules, integrins, as well as other types of activating or inhibitor receptors. This finding is in agreement with previous studies that show that MNPs may not significantly affect expression levels of surface markers in a wide variety of immune cells (38, 39, 72, 80–82).

Another aspect that has been assessed in this work is the degranulation capacity of NK cells after MNP treatment. As mentioned previously, the presence of the functional marker CD107a increases in the cytolytic cells once they have been activated and have released their granules, which contain the enzymes that will lyse the target cells. Therefore, it is an indirect measure of cytolytic capacity (59) and can be produced either by activation in the presence of target cells or through cross-linking with ligands of activating receptors (62, 63). Our analysis of degranulation capacity in NK cells, both in the primary murine NK cells and in the NK-92MI cell line, showed a dose-dependent increase in spontaneous degranulation, that is, in the absence of stimuli, while degranulation in the presence of different stimuli probably increased only because of the increase in basal levels. This could be due to some non-specific activation of the NK cells by the APS-MNPs.

One of the key steps required for an effector NK cell to exert its cytolytic activity against malignant cells is the physical interaction between both cell types (60), a phenomenon known as conjugation. The dynamics of conjugate formation between primary murine NK cells or NK-92MI cells with their corresponding target cells did not appear to be affected by the presence of the APS-MNPs in the NK cell membrane. These results verify, therefore, that the presence of APS-MNPs on the cell surface of cytotoxic cells does not affect their ability to physically interact with and adhere to cells susceptible to being lysed so that NK cells can recognize them and be subsequently activated.

As we have seen, the MNPs do not seem to negatively affect the conjugation and degranulation capacities of the NK and T cells, and thus their direct cytotoxic capacity was subsequently evaluated against several targets. The cytotoxicity of NK cells, when induced by such cell targets that present null or very low levels of MHC-I as RMA/S (against primary murine NK cells) or K562 (against the NK-92MI cell line) or the activating receptor NG2D ligand, YAC-1 (against primary murine NK cells), was not affected by the presence of MNPs. Both murine and human NK cells are capable of being activated and producing lysis of target cells *in vitro*, even in association with MNPs, as previously described (30, 72–74, 76–79).

In addition, the ability to produce IFN-γ after the introduction of various stimuli was also evaluated, as this is one of the most important pro-inflammatory cytokines that produce this type of cells (8). Both murine and human NK cells were able to produce IFN-γ after activation by a variety of non-specific stimuli such as PMA and ionomycin, as well as specific stimuli such as target cells (K562 cells) or the cross-linking of activating receptors (NK1.1, NKG2D, and NKp46) (62, 63), doing so at similar levels in the presence or absence of increasing concentration of MNPs. These findings are similar to previously obtained results (67, 70, 71). However, an increase was observed in the percentage of NK-92MI cells able to produce IFN-γ after non-specific stimulation with PMA and ionomycin when the highest dose of MNPs was used, although a more exhaustive analysis would be necessary to understand the cause of this increase. It is important to note that NKG2D activation is able to stimulate IFN-γ production in murine but not in human NK cells (83, 84), and as a result the specific production of IFN-γ in NK-92MI cells was only analyzed by measuring activation in the presence of target cells (K562 cells).

It is also relevant that these cells with antitumor activity are continuously circulating and recruited from the bloodstream to the tumor through different processes (85, 86). Therefore, we also evaluated the capacity of primary murine NK cells to adhere to an endothelium as well as their transmigration capacity through an endothelium monolayer, finding no defects in this capacity. We observed that the presence of MNPs in the membrane of the cells could occasionally increase their adhesion to endothelial cells. Hypothetically, this may be because these MNPs exposed in the membrane interacted with the endothelial cells. The results obtained in this research are consistent with several studies that showed that NK cells associated with MNPs could migrate and infiltrate the tumor *in vivo* without difficulty (72–74, 76–79).

Chemotactic gradients are also essential to recruit NK cells to the different regions where they are required. When associated with MNPs, the two models used in this study showed no significant alteration in their chemotactic response. However, the application of an EMF slightly increased the migration of these APS-MNP-loaded murine and human NK cells toward their respective chemotactic gradients. This EMF could facilitate the migration of these APS-MNP-loaded cells to the chemotactic gradient, as previously reported (43).

After evaluating different effector functions of the NK cells, and having observed that the MNPs did not cause alterations or defects in these cells, which can maintain their functionality more or less intact in the presence of MNPs, we proceeded to evaluate whether the magnetic retention of these APS-MNP-loaded NK cells could be achieved *in vitro* in the presence of flow forces similar to the ones in the capillaries. This could have potential for subsequent *in vivo* application. Here, the magnetic retention of both murine and human NK cells associated with increasing doses of APS-MNPs was evaluated in a dynamic flow system using magnets with different magnetic forces. The displacement of APS-MNP-loaded murine or human NK cells toward the EMF obtained was similar to the one observed in a previous study using murine and human T cells (43). These results confirmed that this magnetic retention increases with the amount of MNPs linked to the cells as well as with increasing levels of magnetic force.

Our findings lend further support for the use of an EMF to promote *in vitro* retention of lymphoid cells such as NK cells when associated with APS-MNPs and in the presence of flow forces. Previous studies have shown that MNPs or MNP-loaded cells can be injected systemically and targeted to a certain region in mice by applying an EMF such as an external magnet (87–90). Nevertheless, this could only be applied to superficial areas as well as in a model with a reduced size such as mice. An alternative approach to address these questions could be magnetic resonance targeting (MRT). Some studies showed that MRI systems could be used to simultaneously improve cell targeting and tracking (29, 91) and probably help accumulate cells to specific regions like metastatic lesions. This could be of great relevance for NK cell targeting to metastasis, as high ratio of NK cells to tumor cells can be effective for eradicating these lesions. In this regard, Muthana et al. published a study about the potential of MRI scanners to target one or more tissues (91). In this work, they were able to drive MNP-loaded human macrophages to prostatic tumors in mice. Another work recently showed successful MRI guidance of MNP-loaded T cells to tumor sites (29). These results encourage the use of clinical MRI scanners to promote magnetic targeting in deep tissues.

## Conclusions

Using a range of *in vitro* studies, this study shows that the attachment of APS-MNPs to the surface of different models of NK cells did not crucially affect the functionality of these cytolytic cells. Furthermore, the magnetic retention of these APS-MNP-loaded murine and human NK cells was successfully achieved, thereby confirming the potential of this approach to promote the specific accumulation of these cytolytic cells *in vivo*. This combined therapy could ensure that a larger number of totally functional NK cells reaches the tumor, leading to more efficient treatment and better clinical outcomes. This knowledge could be used to design more effective treatment protocols.

## Data Availability

The datasets generated for this study are available on request to the corresponding author.

## Author Contributions

DB, JR, and LS-O designed the study and analyzed the data. LS-O and JR conducted the experiments. YP and SP-Y assisted with some experiments and gave technical support. DB, JR, and LS-O prepared and revised the manuscript, the final version of which was read and approved by all.

### Conflict of Interest Statement

The authors declare that the research was conducted in the absence of any commercial or financial relationships that could be construed as a potential conflict of interest.
