# Supplementary material for: Magnetic Nanoparticles Attached to the NK Cell Surface for Tumor Targeting in Adoptive Transfer Therapies Does Not Affect Cellular Effector Functions
Source: Front Immunol. 2019 Aug 30;10:2073. doi: 10.3389/fimmu.2019.02073 (PMC6728794; doi:10.3389/fimmu.2019.02073)
Supplement: Supplementary file 1 [file Data_Sheet_1.docx]

**SUPPLEMENTARY MATERIAL**

**SUPPLEMENTARY FIGURES AND TABLES**

| **Name** | **Coating** | **Core diameter (nm)** | **Hydrodynamic diameter (nm)** | | **Organic composition (%)** | **Z-potential (mV)** |
| --- | --- | --- | --- | --- | --- | --- |
| **DMSA-MNP** | dimercaptosuccinic  acid | 12.5 ± 2.6 | 83 | 10 | | -34 |
| **APS-MNP** | 3-aminopropyl-triethoxysilane |  | 82 | 10 | | +38 |
| **DEXT-MNP** | dextran 6 kDa |  | 119 | 38 | | -2 |

**Table S1.** Characterisation of DMSA-MNPs, APS-MNPs, and DEXT-MNPs.


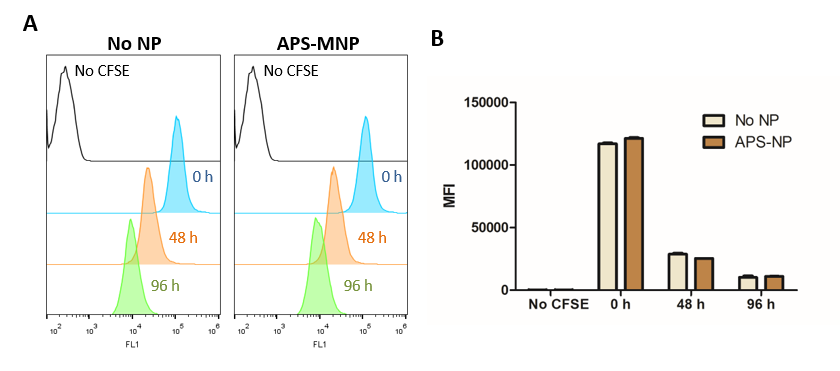


**Figure S1. APS-MNP did not affect NK-92MI growth.** NK-92MI cells were labelled with CFSE and proliferation was measured at 0, 48 and 96 h by flow cytometry in cells left untreated (No NP) or loaded with 150 μg/mL APS-MNP. (A) Representative histograms of CFSE-labelled NK-92MI cells are shown. (B) Mean fluorescence intensity (MFI) (Mean ± SD in 2 experiments) of CFSE signal in untreated or APS-MNP loaded cells are plotted. No differences in loss of CFSE signal (indicative of cell division) was detected between untreated and APS-MNP-loaded cells (two-way ANOVA with Bonferroni post-test).

**
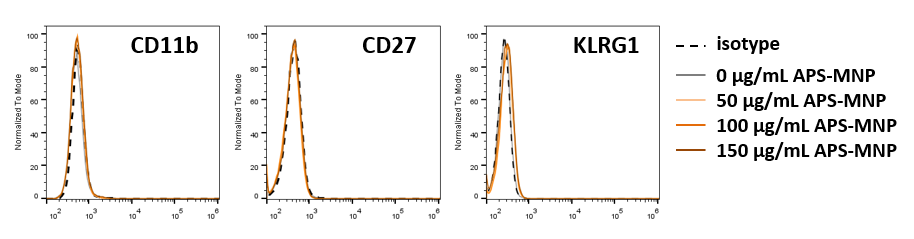
**

**Figure S2. NK-92MI cells do not express CD11b, CD27 or KLRG1 and APS-MNP treatment did not induce the expression of these markers.** Representative histograms of CD11b, CD27 and KLRG1 staining in NK-92MI cells untreated (0 μg/mL) or treated with increasing concentration of APS-MNP and isotype control staining. Expression of these markers was not detected in NK-92MI cells independently of the presence of APS-MNP.

**
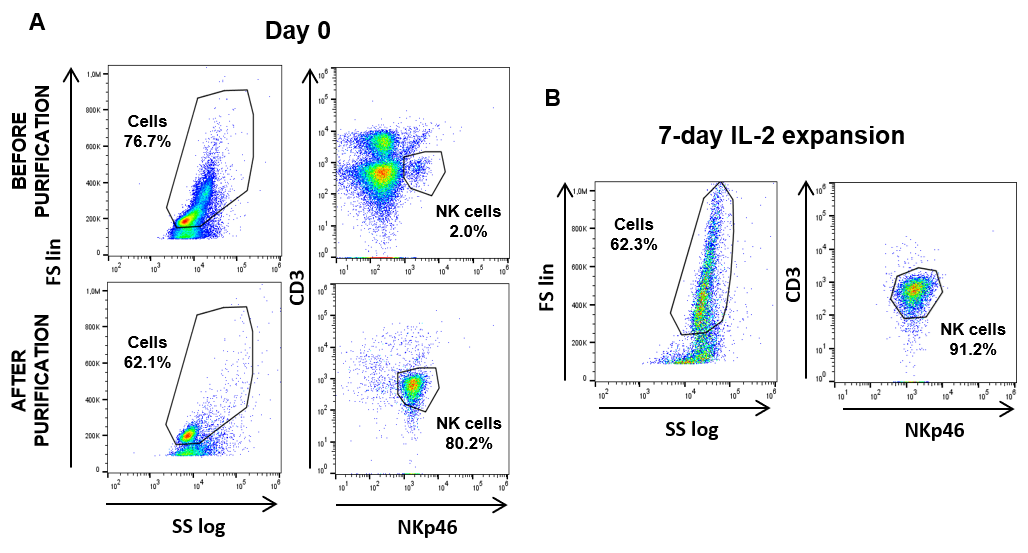
**

**Figure S3. Primary murine NK** **cell purification and expansion.** Representative graphs obtained using FlowJo software from the NK cell population (CD3^-^NKp46^+^ cells) at (A) day 0, before and after NK cell purification, and at (B) day 7, following IL-2 expansion.
